# Supplementary material for: Playability and Player Experience in Digital Games for Elderly: A Systematic Literature Review
Source: Sensors (Basel). 2020 Jul 16;20(14):3958. doi: 10.3390/s20143958 (PMC7411672; doi:10.3390/s20143958)
Supplement: Supplementary file 1 [file sensors-20-03958-s001.pdf]

Supplementary Material.

Tables S-1 (documents based on case studies), S-2 (documents based on proposals, without empirical data), S-3 (revisions), and S-4 (contribution focus) are shown below.

Table S-1: Documents based on case studies (2nd selection, with orange color)**:**

Authors	Article Title	Publication Year	DB Digital	Document Type	Source
Kniestedt, I. Lukosch, S. Brazier, F.	User-centered design of an online mobile game suite to affect well-being of older adults [28]	2018	SPRINGER OTRO	Conference paper	17th IFIP TC 14 International Conference paper: Entertainment Computing – ICEC 2018
Merilampi, S. Koivisto, A. Virkki, J.	Activation game for older adults - development and initial user experiences [80]	2018	SCOPUS SCHOLAR GOO	Conference paper	6th Internat. Conference paper on Seiuos Games and Applications for Health SeGAH 2018
Kappen, Dennis Mirza-Babaei, P. Nacke, Lennart E.	Gamification of Older Adults´ Physical activity: An eight-week study [87]	2018	SPRINGER	Conference paper	Proceedings of the 51 Hawaii Internat. Conference paper on System Sciences
W. de Vries, A. Van Dieen, J. Van den, V. Verschueren, S.	Understanding motivations and player experiences of older adults in virtual reality training [74]	2018	OTRO SCHOLAR GOO	Journal paper	GAMES FOR HEALTH JOURNAL: Research, Development, and Clinical Applications, Vol. 7, N° 6;
Johnson, D. Gardner, M. J. Perry, R.	Validation of two game experience scales: the player experience of need satisfaction (PENS) and game expe-rience questionnaire (GEQ) [32]	2018	SCIENCE DIRECT OTRO	Journal paper	International Journal of Human-Computer Studies; 118; Pps 38-46.
Brox, E. Kontantinidis, S. Evertsen, G.	User-centered design of serious games for older adults following 3 years of experience with exergames for seniors: a Study design [27]	2017	OTRO SCHOLAR GOO	Journal paper	JMIR Seriou Games; 2017.
Pyae, A. Liukkonen, T.N. Mika, L. Kattimeri, C.	Investigating the Finnish elderly people´s user experience in playing digital	2017	SCOPUS	Journal paper	Gerontechnology, Vol. 16, N° 2.

	game-based skiing exercise: a usability study [49]				
Altmeyer, M. Lessel, P.	The important of social relations for well-being change in old age - Do games preferences change as well? [64]	2017	OTRO	Conference paper	Positive Gaming: Workshop on Gamification and Games for Wellbeing, CHI PLAY '17; Amsterdam, October 15,
Birk, M. Friehs, M. A. Mandryk, R. L.	Age-based preferences and player experience: a crowdsourced cross-sectional study [15]	2017	ACM OTRO	Conference paper	Proceedings of the Annual Symposium on Computer-Human Interaction in Play; Pages 157-170 October 15–18.
Palacio, R. Acosta, C. O. Cortez, J. Morán, A.	Usability perception of different video game devices in elderly users [94]	2017	SPRINGER OTRO	Journal paper	Journal Universal Access in the Information Society, Vol. 16 N° 1, March 2017, Pages 103-113
Pyae, A. Liukkonen, T.N. Luimula, M. Smed, J.	Investigation the Finnish elderly people´s attitudes and motivation towards digital game-based physical exercises [106]	2017	SCHOLAR GOO	Journal paper	Finnish Journal of eHealth and eWelfare, Vol 9 No 4; November 2017.
Wang, X. Salehzadeh, K. Ren, X. Wang, Z.	How Skill balancing impact the elderly player experience? [2]	2016	IEEE OTRO	Conference paper	13th International Conference paper on Signal processing (ICSP); 6-10 Noviembre, Chengdu, China.
Sayago, S. Rosales, A. Righi, V. Coleman, G. W.	On the conceptualization, design, and evaluation of appealing, meaningful, and playable digital games for older people [47]	2016	SCHOLAR GOO	Journal paper	Games and Culture, Vol. 11 (1-2); SAGE.
Pyae, A. Raitoharju, R. Luimula, M. Smed, J.	Serious games and active ageing: a pilot usability testing of existing games [63]	2016	SCOPUS SCHOLAR GOO	Journal paper	International Journal of Networking and Virtual Organisations; Vol.16 N° 1.
Boletsis, C. McCallum, S.	Evaluating a gaming system for cognitive screening and sleep duration assessment of elderly players: a pilot study [86]	2016	SCOPUS	Conference paper	GALA 2016 - International Conference paper on Games and Learning Alliance; November, pp. 107-119.

Brown, J.<br>Zhou,J.	Exploring the next generation of older gamers: middle-aged gamers [107]	2016	SPRINGER<br>SCHOLAR<br>GOO	Conference paper	ITAP International Conference paper on Human Aspects of IT for the Aged Population, Part II, July 2016.
Liukkonen, T.N.<br>Makila, T.<br>Ahtosalo, H<br>Raitohatju, R.	Motion Tracking exergames for elderly users [77]	2015	OTRO<br>SCHOLAR<br>GOO	Journal paper	IADIS International Journal on Computer Science & Information Systems; Vol.10
Pyae, A.<br>Luimula, M<br>Smed, J.	Investigating the usability of interactive physical activity games for elderly: a pilot study [82]	2015	SCOPUS<br>SCHOLAR<br>GOO	Conference paper	CoginfoCom 2015; Proceedings of the IEEE 6th Conference paper on Cognitive Infocommunications; October,
Wagner, I.<br>Minge, M.	The gods play dice together: the influence of social elements of gamificaction on seniors´user experience [85]	2015	SCOPUS<br>SCHOLAR<br>GOO	Conference paper	HCI International 2015. Posters' Extended Abstracts, Part I; Berlin, August.
Saenz-Urturi, Z.<br>García Z., B.<br>Méndez Z., A.	Elderly user experience to improve a Kinect-based game playability [60]	2015	SCOPUS<br>WOS<br>SCHOLAR<br>GOO	Journal paper	Behaviour and Information Technology 34(11); October.
Paavilainen, J.<br>Alha, K.<br>Korhonen, H.	Domain-specific playability problems in social networks games [56]	2015	OTRO<br>SCHOLAR<br>GOO	Journal paper	International Journal Arts and Technology, Vol. 8, No. 4, 2015.
Sopanen, J.	Elderly clients´experiences of playing a video-game [100]	2015	OTRO<br>SCHOLAR<br>GOO	Thesis	Bachelor's Thesis, Degree Programme in Nursing; Laurea University of Applied Sciences; Otaniemi, Finland.
Koivisto, A.<br>Merilampi, S.<br>Sirkka, A.	Mobile rehabi-litation games - user experience study [78]	2014	SCOPUS<br>SCHOLAR<br>GOO	Conference paper	European Conference paper on Games-based learning 2
Genaro, L.<br>Vigouroux, N<br>Gorce, P.	Drag-and-drop for older adults using Touchscreen devices: effects of screen sizes and interaction techniques on accuracy [88]	2014	SCOPUS	Conference paper	IHM´14 26th Conférence francophone sur lÍnteraction Homme-Machine; Oct 2014
Wang, J-Y.	Comparision of game experience and preferences between young and elderly [58]	2014	IEEE<br>OTRO	Conference paper	ICALIP2014; International Conference paper on Audio, Language and Image Processing

Awad, M. Fergurson, S. Craig, C.	Designing games for older adults: an affordance based approach [92]	2014	IEEE OTRO	Conference paper	IEEE International Conference paper on Serious Games and Applications for Health (SeGAH); Held 14-16 May.
Nawaz, A. Skjaeret, N. Ystmark, K. Vereijken, B.	Assessing seniors´user experience (UX) of exergames for balance training [61]	2014	ACM OTRO	Conference paper	NordiCHI '14 Proceedings of the 8th Nordic Conference paper on Human-Computer Interaction: Fun, Fast, Foundational; Helsinki, Finland; October 26 - 30
Rodriguez, J. Palacio, R. Acosta, C. Grimaldo, A.	Los videojuegos como actividad de ocio en adultos mayores: la experiencia de un grupo focal [31]	2014	OTRO SCHOLAR GOO	Conference paper	Memoria del Encuentro Nacional de Ciencias de la Computación - ENC 2014; 3-5 de Noviembre NovaUniversitas, Oaxaca, México.
Marston, H.	Design recommendations for digital game design within an ageing society [62]	2013	OTRO SCHOLAR GOO	Journal paper	International Journal of Human-Computer Studies, Vol. 108; December.
Smeddinck, J. Gerling, K. Tiemkeo, S.	Visual complexity, player experience, performance and physical exertion in motion-based games for older adults [59]	2013	ACM OTRO	Conference paper	ASSETS '13 Proceedings of the 15th International ACM SIGACCESS Conference paper on Computers and Accessibility; Art. No. 25,
Satoshi, A. L. Farinazzo, V. Da Silva, F.	Usability evaluation of an application designed for the older adults [96]	2013	SPRINGER OTRO	Conference paper	ICEC 2013, International Conference paper on Entertainment Computing;
Patsoule, E. Koutsabasis, P.	Redesigning web sites for older adults: a case study [81]	2012	OTRO SCHOLAR GOO	Conference paper	5ta Internac. Conference paper of Pervasive Technologies PETRA´12
Pham, T. P. Theng, Y-L.	Game controllers for older adults: experimental study on gameplay experiences and preferences [83]	2012	ACM SCHOLAR GOO	Conference paper	FDG '12 Proceedings of the International Conference paper on the Foundations of Digital Games; Raleigh, North Carolina
Diaz-Orueta, U. Facal, D. Nap, H. H. Ranga, M-M.	What is the key for older people to show interest in playing digital learning games? Initial qualitative findings from the LEAGE project on a multicultural	2012	OTRO SCHOLAR GOO	Journal paper	Games for Health Journal: Research, Development, and Clinical Applications, Vol. 1, N° 2

	european sample [93]				
Gerling, K.<br>Schild, J.<br>Masuch, M.	Exergaming for elderly: analyzing player experience and performance [33]	2012	OTRO<br>SCHOLAR<br>GOO	Journal paper	Mensch & Computer 2011;
Brown, J.	Let´s Play: Understanding the role and Meaning of Digital Games in the lives of Older Adults [103]	2012	SCHOLAR<br>GOO	Conference paper	CFP: Foundations of Digital Games 2012, May 29 - June 1, Raleigh, NORTH CAROLINA.
Gonzalez, J. L.<br>Padilla, N.<br>Gutierrez, F.<br>Montero, F.	Jugabilidad como calidad de la Experiencia del Jugador en videojuegos [55]	2012	SCHOLAR<br>GOO<br>OTRO	Conference paper	CoSECivi 2014 - Sociedad Española para las Ciencias del Videojuego.
Mahmud, A.<br>Shahid, S.<br>Mubin, O.	Designing with and for older adults: experience from gane design [105]	2012	SPRINGER<br>SCHOLAR<br>GOO	Journal paper	Human-Computer Interaction, The Agency Perspective ; pp. 111–129
Gerling, K.<br>Schulte, F.<br>Masuch, M.	Designing and evaluation digital games for frail elderly persons [90].	2011	OTRO	Conference paper	ACE '11 Proceedings of the 8th International Conference paper on Advances in Computer Entertainment Technology; Art. No. 62
Gonzalez, J. L.<br>Gil, Rosa<br>Gutierrez, F.	Enriching evaluation in video games [53]	2011	OTRO	Conference paper	Proceedings of the 13th IFIP TC 13 international Conference paper on Human-Computer Interaction - Volume Part IV.
Gajadhar, B.<br>Nap, H. H.<br>De Kort, Y.<br>Ijsselsteijn	Out of sight, out of mind: co-player effects on seniors' player experience [14]	2010	ACM<br>SCHOLAR<br>GOO	Conference paper	3rd International Conference paper on Fun and Games; Leuven, September 15-17
Nacke, L.<br>Nacke, Anne<br>Lindley, C.A.	Brain Training for Silver Gamers: effects of age and game form on effectiveness, efficiency, self-assessment, and gameplay experience [102]	2009	SCHOLAR<br>GOO<br>OTRO	Journal paper	CYBERPSYCHOLOGY & BEHAVIOR, Vol. 12, N° 5, September

Shahid, S.<br>Mubin, O.<br>Mahmud, A.	RACE: towards exploring the design dimensions of a route assisting and communicating system for elderly [108]	2009	SPRINGER<br>SCHOLAR<br>GOO	Conference paper	UAHCI '09 - 5th International Confer. paper on Universal Access in Human-Computer Interaction. Addressing Diversity. Part I; San Diego, CA, July 19 – 24.
Nap, H.<br>de Kort, Y.<br>Ijsselsteijn, W.	Senior gamers: Preferences, motivations and needs [51]	2009	SCHOLAR<br>GOO<br>OTRO	Journal paper	Gerontechnology, Vol. 8, No 4; October.
Mahmud, A.<br>Mubin, O.<br>Shahid, S.<br>Martens, J.B.	Designing and Evaluating the Tabletop Game Experience for Senior Citizens [101]	2008	ACM<br>SCHOLAR<br>GOO	Conference paper	NordiCHI '08 Proceedings of the 5th Nordic Conference paper on Human-Computer Interaction: building bridges; Lund, Sweden, Oct 20 – 22
Cabrera, M.E.<br>Ferrer, M.C.<br>Romero, M.T.<br>Poveda, R.	Juego como promoción de un envejecimiento saludable. [99]	2006	SCIENCE<br>DIRECT<br>OTRO	Journal paper	Revista Española de Geriatría y Gerontología; Volume 41, Supplement 2.

Table S-2: Documents without empirical data (proposals):

Authors	Article Title	Publication Year	DB Digital	Document Type	Source
Fanfarelli, J.<br>McDaniel, R.<br>Crossley, C.	Adapting UX to the design of health-care games and applications [75]	2018	SCIENCE<br>DIRECT	Journal paper	Entertainment Computing, Vol. 28, December
Aleem, S.<br>Capretz, L. F.<br>Ahmed, F.	A consumer perspective on digital games [89]	2018	IEEE<br>OTRO	Journal paper	IEEE Consumer Electronics Magazine; May 2018
Pyae, A.	Investigating the usability, user experiences, and usefulness of digital game-based exercises for elderly people: a case study of Finland [52]	2018	ACM<br>OTRO	Conference paper	CHI PLAY´18 Annual Symposium on Computer-Human Interaction in Play Companion Extended Abstracts; October 28–31.
Righi, V.<br>Sayago, S.<br>Blat, J.	When we talk about older people in HCI, who are we talking about? Towards a 'turn community' in the design of technologies for a growing ageing population [46]	2017	SCHOLAR<br>GOO	Journal paper	International Conference paper on Digital interactive media in entertainment and arts (DIMEA); Perth, Australia.

Paavilainen, J.	Playability: A game-centric definition [40]	2017	SCHOLAR GOO	Conference paper	Annual Symposium on Computer-Human Interaction in Play, CHI PLAY´17; October 15-18, Amsterdam.
Awad, M. Craig, C.	Player´s performance in cross generational game playing [74]	2017	SCOPUS SPRINGER	Conference paper	Third Joint International Conference paper, JCSG 2017, Valencia; November 23-24.
Korn, Oliver Tietz, Stefan	Strategies for Playful design when gamifying rehabilitation: a study on user experience [76]	2017	ACM SCHOLAR GOO	Conference paper	10th Int. Conference paper on Pervasive Technologies Related to Assistive Environments, PETRA '17,
Segura, E.M., Waern, A. Márquez, Luis López, David	Playification: The PhySeEr case [79]	2016	SCHOLAR GOO	Conference paper	Proceedings of the 2016 Annual Symposium on Human-Computer Interaction in Play Pps. 376-388
Silva, P. A. Holden, Kelly Jordan, P.	Towards a List of heuristics to Evaluate Smartphone Apps targeted at Older Adults: A study with Apps taht Aim at promoting Health and Well-being [65]	2015	IEEE SCHOLAR GOO	Conference paper	48th Hawaii International Conference paper on System Sciences.
Teixeira C., T. Ishitani, L. Vieira, N.	Mobile game design for the elderly: a study with focus on the motivation to play [71]	2015	OTRO	Journal paper	Computers in Human Behavior 51; Pps. 96-105.
Mainza, E.	HCI: design guidelines of mobile device games for the elderly [95]	2014	OTRO SCHOLAR GOO	Thesis	Master Thesis, Faculty of Technology and Society: Dept. of Computer Science; Malmo University.
Novick, D. Vicario, J. Santaella, B. Gris, I.	Empirical Analysis of Playability vs. Usability in a computer game [109]	2014	SPRINGER SCHOLAR GOO	Conference paper	Third International Conference paper on Design, User Experience, and Usability. User Experience Design for

					Diverse Intera-tion Platforms and Environments, Part II; June 22-27.
McLaughlin, A. C.<br>Bryant, M.<br>Sprufera, J.<br>Gandy, M.	Usability an important goal for the design of therapeutic games for older adults [91]	2013	SPRINGER<br>OTRO	Conference paper	Proceedings of the 10th international Conference paper on Engineering Psychology and Cognitive Ergonomics: applications and services - Volume Part II
Ibrahim, A.<br>Gutierrez, F.<br>Gonzalez, J.L.<br>Padilla, N.	Educational Playability: Analyzing Player Experience in Educational Video Games [72]	2012	OTRO<br>SCHOLAR<br>GOO	Conference paper	ACHI 2012: The Fifth International Conference paper on Advances in Computer-Human Interactions; Febrary, Valencia, España.
Sanchez, J. L.<br>Gutierrez, F.<br>Montero, F.<br>Padilla, N.	Playability: analysing user experience in video games [41]	2012	OTRO<br>SCHOLAR<br>GOO	Journal paper	Behaviour & Information Technology Vol. 31, No. 10, October 2012, 1033–1054
Blat, J.<br>lluis, J.<br>Sayago, S.	WorthPlay: Juegos Digitales para un envejecimiento activo y saludable [97]	2012	OTRO<br>SCHOLAR<br>GOO	Journal paper	Cuadernos de la Fundación General CSIC, Nº 8.
Cámara, A.	El Juego en las Personas Mayores: una vía de desarrollo personal [98]	2012	OTRO<br>SCHOLAR<br>GOO	Journal paper	Revista Portuguesa de Pedagogía; N° 46-I, 2012.
Nacke, L.<br>Drachen, A.	Towards a framework of player experience research [12]	2011	ACM<br>OTRO	Conference paper	Proceedings EPEX´11; Julio; Bordeaux, Francia
Nacke, L.<br>Drachen, A.<br>Poels, K.<br>Ijsselsteijn	Playability and player experience research [13]	2009	OTRO<br>SCHOLAR<br>GOO	Conference paper	DiGRA International Conference paper: Breaking New Ground: Innova-tion in Games, Play, Practice and Theory; Septiembre.

Gonzalez, J.L. Padilla, N. Gutierrez, F.	From Usability to Playability: Introduction to Player- Centred Video game Development process [43]	2009	SPRINGER OTRO	Conference paper	Proceedings of the 1st International Conference paper on Human Centered Design; San Diego, CA — July 19 - 24
Sanchez, J. L. Padilla, N. Gutierrez, F.	Playability: How to identify the Player Experience in a video game [42]	2009	SPRINGER SCHOLAR GOO	Conference paper	INTERACT 2009 - Human-Computer Interaction, Part I; Agosto
Nacke, L. Ambinder, M. Canossa, A. Stach, T.	Game metrics & biometrics [104]	2009	OTRO SCHOLAR GOO	Conference paper	Panel at Future Play; May 12-13, 2009, Vancouver, Canada.
Gonzalez, J. L. Padilla, N. Gutierrez, F. Cabrera, M.J.	De la Usabilidad a la Jugabilidad: Diseño de videojuegos centrado en el Jugador [10]	2008	OTRO SCHOLAR GOO	Conference paper	Actas de Interacción 2008, pp. 99-109.
Ijsselsteijn Nap, H. H. De Kort, Y. Poels, K.	Digital Game Design for Elderly Users [48]	2007	ACM	Conference paper	Proceedings of the 2007 Conference paper on Future Play; Págs. 17-22; Toronto, Canada; November.
Desurvire, H. Caplan, M. Toth, J.	Using heuristics to evaluate the playability of games [57]	2004	ACM SCHOLAR GOO	Conference paper	CHI '04 extended abstracts on Human factors in computing systems; Vienna, Austria.

Table S-3: Reviews:

Authors	Article Title	Publication Year	DB Digital	Publication Year	Source
Wang, J.	Digital Game Design for Elderly Users: A Multi-Disciplinary Review [26]	2016	OTRO SCHOLAR GOO	Journal paper	School of Computer Science, The University of Birmingham; August.
Nawaz, A. Skjaeret, N. Boulton, E. Vereijken, B.	Usability and acceptability of balance exergames in older adults: a scoping review [84]	2015	SCOPUS SCHOLAR GOO	Journal paper	Health Informatics Journal, Vol. 22(4), pps. 911-931; August 2015.

Teixeira, T. Ishitani, L.	Motivation and benefits of digital games for the elderly: a systematic literature review [50]	2015	SCHOLAR GOO	Journal paper	Revista Brasileira de Computação Aplicada (ISSN 2176-6649), Passo Fundo, Vol. 7, n. 1, p. 2-16.
Caroux, L. Isbister, K. Le Bigot, L. Vibert, N.	Player-video game interaction: a systematic review of current concepts [110]	2015	SCIENCE DIRECT OTRO	Journal paper	Computers in Human Behavior · July 2015.

Table S-4: Article grouped by contribution Focus:

CONCEPT	CONTENTS	ANALIZED DOCUMENTS
Playability<br><br>Total: 16	The set of properties that describe the player's experience before a specific game system, whose main objective is to entertain and entertain in a satisfactory and credible way, when playing alone or accompanied. It describes the quality of the judge, in terms of its rules of operation and its design; corresponds to what the player does during the game.	Case Studies: [2] [28] [49] [53] [55] [56]<br>Proposals: [10] [13] [40] [41] [42] [43] [52] [57] [89] [109]
Player Experience<br><br>Total: 28	Investigate the emotional, cognitive and social components of the experience that arises from the interaction between the players and a game system. It is the user experience that occurs while the player interacts with the game.	Case Studies: [14] [15] [27] [33] [49] [53] [58] [59] [60] [61] [62] [74] [80] [82] [83] [85] [86] [101] [102]<br>Proposals: [12] [13] [41] [52] [72] [75] [76] [95] [104]
Digital Games for Elderly<br><br>Total: 36	Publications that teach how digital games provide opportunities to improve the quality of life of the elderly. They present experiences in which digital games are a promising technology that can improve the quality of life in terms of physical, social and cognitive well-being. They do not include discussions or conclusions about specific aspects of playability and player experience.	Case Studies: [31] [32] [47] [51] [63] [64] [77] [78] [81] [87] [88] [90] [92] [93] [94] [96] [99] [100] [103] [105] [106] [107] [108]<br>Proposals: [46] [48] [65] [71] [79] [91] [97] [98] [111]<br>Reviews: [26] [50] [84] [110]

References

1. Pyae, A. The potential of digital games in promoting older people's active ageing in developing countries: The case of Myanmar. *Int. J. Educ. Ageing* **2017**, 4, 19–34.
2. Wang, X.; Niksirat, K.S.; Silpasuwanchai, C.; Wang, Z.; Ren, X.; Niu, Z. How skill balancing impact the elderly player experience? In Proceedings of the 2016 IEEE 13th International Conference on Signal Processing (ICSP), Chengdu, China; 6–10 November 2016; pp. 983–988; doi: 10.1109/ICSP.2016.7877976.
3. De Schutter, B.; Abeele, V.V. Meaningful play in elderly life. In Proceedings of the Fifth Australasian Conference on Interactive Entertainment, New York, NY, USA, 3–5 December 2008.
4. Jung, Y.; Li, K.J.; Janissa, N.S.; Gladys, W.L.C.; Lee, K.M. Games for a better life. In Proceedings of the Sixth Australasian Conference, Sydney, Australia, 17–19 December 2009.
5. Csikszentmihalyi, M. *Flow: The Psychology of Optimal Experience*; Harper & Row: New York, NY, USA, 2013.

6. Ascolese, A.; Kiat, J.; Pannese, L.; Morganti, L. Gamifying elderly care: Feasibility of a digital gaming solution for active aging. *Digit. Med.* **2016**, *2*, 157–162.
7. ISO/IEC. Systems and Software Engineering: Systems and Software Quality Requirements and Evaluation (SQuaRE)—System and Software Quality Models. Avalaible online: https://www.iso.org/obp/ui/#iso:std:iso-iec:25010:ed-1:v1:en (access on 2 April 2019).
8. Ortega, S. Introducción a la Usabilidad y su Evaluación. Mater's Thesis, Universitat Obrtea de Catalunya, La Rioja, Spain, 2010.
9. ISO 9241-210:2019. *Ergonomics of Human-System Interaction—Part 210: Human-Centred Design for Interactive Systems*; Standards Catalogue, International Organization for Standardization: Central Secretariat, Geneva, Switzerland, 2019.
10. González, J.L.; Padilla, N; Gutierrez, L.; Cabrera, M. De la Usabilidad a la Jugabilidad: Diseño de Videojuegos Centrado en el Jugador. In Proceedings of the IX Congreso Internacional Interacción, Albacete, España; 9–11 June 2008; pp. 99–109
11. Gonzalez, J.L. Jugabilidad: Caracterización de la Experiencia del Jugador en Videojuegos. Ph.D. Thesis, Universidad de Granada, Granada, Spain, 2018.
12. Nacke, L.; Drachen, A. Towards a framework of player experience research. In Proceedings of the EPEX´11, Bordeaux, France, 29 June–1 July 2011.
13. Nacke, L., Drachen, A.; Kuikkaniemi, K.;Niesenhaus, J.; Korhonen, H.J.; Hoogen, W.M.v.d.; Poels, K.; Ijsselsteijn, W.A.; De Kort, Y.A. Playability and Player Experience Research. In Proceedings of the 2009 DiGRA International Conference: Breaking New Ground: Innovation in Games, Play, Practice and Theory, London, UK, 1–4 September 2009
14. Gajadhar, B.; Nap, H.; De Kort, Y.A.; IJsselsteijn, W.A. Out of sight, out of mind: Co-player effects on seniors' player experience. In Proceedings of the 3rd International Conference on Fun and Games, Leuven, Belgium,15–17 September 2010. pp 74–83.
15. Birk, M.V.; Friehs, M.A.; Mandryk, R.L.; Schouten, B.; Markopoulos, P.; Toups, Z.; Cairns, P.; Bekker, T. Age-Based Preferences and Player Experience. In Proceedings of the Annual Symposium on Computer-Human Interaction in Play—CHI PLAY'17, Amsterdam, The Netherlands, 15–18 October 2017; pp. 157–170.
16. WHO. World Report on Ageing and Health 2015. Available online: http://www.who.int/ageing/events/world-report-2015-launch/en/ (accessed on 10 October 2019).
17. SENAMA. Censo 2017 Reveló que más del 16% de la Población Chilena es Adulto Mayor. Available online: http://www.senama.gob.cl/noticias/censo-2017-revelo-que-mas-del-16-de-la-poblacion-chilena-es-adulto-mayor(accessed on 27 December 2017).
18. Boj, C.; Díaz, D.J.; Portalés, C.; Casas, S. Video Games and Outdoor Physical Activity for the Elderly: Applications of the HybridPLAY Technology. *Appl. Sci.* **2018**, *8*, 1912, doi: 10.3390/app8101912.
19. United Nations. World Population Ageing Report. Available online: http://www.un.org/en/development/ desa/population/publications/pdf/ageing/WorldPopulationAgeing2013.pdf (accessed on 25 July 2019).
20. Staff OMS. Serie Envejecimiento y Ciclo de Vida. Available online: http://www.who.int/ageing/about/facts/es/ (accessed on 14 August 2019).
21. Fondo de Población de Naciones Unidas (UNFPA) y Help Age International: "Envejecimiento en el Siglo XXI: Una Celebración y un Desafío. Resumen Ejecutivo. Nueva York, 2012. Available online: https://www.unfpa.org/sites/default/files/pub-pdf/Ageing%20Report%20Executive%20Summary%20SPANISH%20Final_0.pdf (accessed on 15 May 2019).
22. Staff. Dependencia de los Adultos Mayores en Chile. Departamento de Estudios y Desarrollo, Superintendencia de Salud; Departamento de Economía de la Salud-MINSAL División dePlanificación Regional de MIDEPLAN Marzo 2008. Available online: http://www.supersalud.gob.cl/documentacion/666/articles-4471_recurso_1.pdf (accessed on 10 May 2019).
23. Staff. Older People, Technology and Community. Available online: https://www.cisco.com/c/dam/en_us/about/ac79/docs/wp/ps/Report.pdf (accessed on 25 May 2019).

24. Pyae, A.; Gray, H.; Lewis, A. Investigating the Impacts of Digital Games on Elderly People's Active Ageing in Myanmar: A Systematic Literature Review. In Proceedings of the 15th International Conference on Computer Applications, Yangon, Myanmar, 16 February 2017.
25. Pyae, A.; Joelsson, T.N.; Luimula, M.; Saarenpaa, T.; Smed, J. Lessons Learned from the Gamified Solutions in Healthcare Project: Usability Studies of Digital Game-based Physical Exercises for Elderly People. *EAI Endorsed Trans. Serious Games* **2017**, *4*, doi:10.4108/eai.27-12-2017.153511.
26. Wang, J. *Digital Games Design for Elderly Users: A Multi-Disciplinary Review*; The University of Birmingham: Birmingham, UK; 2016.
27. Brox, E.; Konstantinidis, S.T.; Evertsen, G.; Bird, M.-L.; Immonen, M.; Giraud-Carrier, C. User-Centered Design of Serious Games for Older Adults Following 3 Years of Experience With Exergames for Seniors: A Study Design. *JMIR Serious Games* **2017**, *5*, e2, doi:10.2196/games.6254.
28. Kniestedt, I.; Lukosch, S.; Brazier, F. User-Centered Design of an Online Mobile Game Suite to Affect Well-Being of Older Adults. In Proceedings of the Intelligent Tutoring Systems, Poznan, Poland, 17–20 September 2018; pp. 355–361.
29. Melenhorst, A.S. Adopting Communication Technology in Later Life. The Decisive Role of Benefits. Ph.D. Thesis, Eindhoven University of Technology, Eindhoven, The Netherlands, 2002.
30. Eggermont, S.; Vandebosch, H.; Steyaert, S. To-wards the desired future of the elderly and ICT: Policy recommendations based on a dialogue with senior citizens. *Poiesis Prax.* **2006**, *4*, 199–217.
31. Rodriguez, J.; Palacio, R.; Acosta, C.O.; Casillas, R.; Grimaldo, A.I. Los videojuegos como actividad de ocio en adultos mayores: La experiencia de un grupo focal. In Proceedings of the Memoria del Encuentro Nacional de Ciencias de la Computación—ENC, Ocotlán, México, 3–5 Noviembre 2014.
32. Johnson, D.; Gardner, M.J.; Perry, R. Validation of two game experience scales: The Player Experience of Need Satisfaction (PENS) and Game Experience Questionnaire (GEQ). *Int. J. Hum.-Comput. Stud.* **2018**, *118*, 38–46, doi:10.1016/j.ijhcs.2018.05.003.
33. Gerling, K.M.; Schild, J.; Masuch, M. Exergaming for Elderly: Analyzing Player Experience and Performance. *Mensch Comput.* **2011**, *16*, 401–411, doi:10.1524/9783486712742.401.
34. Johnson, D.; Wyeth, P.; Sweetser, P.; Gardner, J. Personality, Genre and video Game Play Experience. In *Proceedings of the 4th International Conference on Fun and Games*; Association for Computing Machinery (ACM): New York, NY, USA, 2012; pp. 117–120.
35. Johnson, D.; Gardner, J. Personality, Motivation and Video Games. In *Proceedings of the 22nd Conference of the Computer-Human Interaction SpecialInterest Group of Australia on Computer-Human Interaction, 22–26 November 2010, Queensland University of Technology, Brisbane, QLD*; Association for Computing Machinery (ACM): New York, NY, USA, 2010; pp. 276–279.
36. Rigby, S.; Ryan, R. *The Player Experience of Need Satisfaction (Pens): An Applied Model and Methodology for Understanding Key Components of the Player Experience*; Immersyve, Inc: Celebration, FL, USA, 2007. Available online: http://www.immersyve.com/?wpdmdl=8283 (accessed on 16 June 2019).
37. Kim, J.H.; Gunn, D.V.; Schuh, E.; Phillips, B.; Pagulayan, R.J.; Wixon, D. Tracking real-time user experience (TRUE). In Proceedings of the Twenty-Sixth Annual CHI Conference on Human Factors in Computing Systems (CHI'08), Florence, Italy, 5–10 April 2008; Association for Computing Machinery (ACM): New York, NY, USA; pp. 443–451.
38. McAuley, E.; Duncan, T.; Tammen, V.V. Psychometric Properties of the Intrinsic Motivation Inventory in a Competitive Sport Setting: A Confirmatory Factor Analysis. *Res. Q. Exerc. Sport* **1989**, *60*, pp. 48–58, doi:10.1080/02701367.1989.10607413.
39. Crawford, J.R.; Henry, J.D. The positive and negative affect schedule (PANAS): Construct validity, measurement properties and normative data in a large non-clinical sample. *Br. J. Clin. Psychol.* **2004**, *43*, 245–265, doi:10.1348/0144665031752934.
40. Paavilainen, J. Playability—A Game-Centric Definition. Extended Abstracts Publication of the Annual Symposium on Computer-Human Interaction in Play. In Proceedings of the CHI PLAY´17, Amsterdam, The Netherlands, 15–18 October 2017; pp. 487–494.
41. Sánchez, J.L.G.; Vela, F.L.G.; Simarro, F.M.; Zea, N.P. Playability: Analysing user experience in video games. *Behav. Inf. Technol.* **2012**, *31*, 1033–1054, doi:10.1080/0144929x.2012.710648.
42. Sánchez, J.L.G.; Zea, N.P.; Gutierrez, F.L. Playability: How to Identify the Player Experience in a Video Game. *Intell. Tutoring Syst.* **2009**, *5726*, 356–359, doi:10.1007/978-3-642-03655-2_39.

43. Gonzalez, J.L.; Padilla, N.; Gutierrez, F. From Usability to Playability: Introduction to Player- Centred Video Game Development Process; Human Centered Design 09. In Proceedings of the 1st Internat. Conference on Human Centered Design; San Diego, CA, USA, 19–25 July 2009. pp. 65–74.
44. García, F.J. Mapeos Sistemáticos de Literatura, revisiones sistemáticas de literatura y benchmarking de programas formativos. In Proceedings of the Conferencia Grupo GRIAL, Monterrey, Mexico, 27–28 November 2017.
45. Yannascoli, S.; Carey, J.; Schenker, M.; Ahn, J.; Baldwin, K. How to Write a Systematic Review: A step-by-step Guide. *Univ. Orthop. J.* **2013**, *23*, 64–69.
46. Righi, V.; Sayago, S.; Blat, J. When we talk about older people in HCI, who are we talking about? Towards a 'turn to community' in the design of technologies for a growing ageing population. *Int. J. Hum.-Comput. Study* **2017**, *108*, 15–31, doi:10.1016/j.ijhcs.2017.06.005.
47. Sayago, S.; Rosales, A.; Righi, V.; Ferreira, S.M.; Coleman, G.W.; Blat, J. On the Conceptualization, Design, and Evaluation of Appealing, Meaningful, and Playable Digital Games for Older People. *Games Cult.* **2015**, *11*, 53–80, doi:10.1177/1555412015597108.
48. IJsselsteijn, W.; Nap, H.H.; de Kort, Y.; Poels, K. Digital Game Design for Elderly Users. In Proceedings of the 2007 Conference on Future Play, Toronto, ON, Canada, 14–17 November 2007; pp. 17–22.
49. Pyae, A.; Liukkonen, T.; Luimula, M.; Smed, J. Investigating the Finnish elderly people's user experience in playing digital game-based skiing exercise: A usability study. *Gerontechnology* **2017**, *16*, 65–80.
50. Cota, T.T.; Ishitani, L. Motivation and benefits of digital games for the elderly: A systematic literature review. *Revist. Brasileira Comput. Apl.* **2015**, *7*, 56–61, doi:10.5335/rbca.2015.4190.
51. Nap, H.; De Kort, Y.A.W.; Ijsselsteijn, W. Senior gamers: Preferences, motivations and needs. *Gerontechnology* **2009**, *8*, 247–263, doi:10.4017/gt.2009.08.04.003.00.
52. Pyae, A. Investigating the Usability, User Experiences, and Usefulness of Digital Game-based Exercises for Elderly People. In Proceedings of the 2018 Annual Symposium on Computer-Human Interaction in Play Companion Extended Abstracts—CHI PLAY'18 Extended Abstracts, Melbourne, Australia; 28–31 October 2018; pp. 71–76.
53. Gonzalez, J.L.; Gil, R.; Gutierrez, F. Enriching evaluation in video games. In Proceedings of the 13th IFIP TC 13 International Conference on Human-Computer Interaction—Vol. 1 Part IV, Lisbon, Portugal, 5–9 September 2011; pp. 519–522.
54. Procci, K.; Singer, A.R.; Levy, K.R.; Bowers, C. Measuring the flow experience of gamers: An evaluation. *Comput Hum. Behav.* **2012**, *28*, 2306–2313. doi: 10.1016/j.chb.2012.06.039.
55. Gonzalez, J.L.;. Padilla, N.; Zea, N.P.; Montero, F. Jugabilidad como Calidad de la Experiencia del Jugador en Videojuegos. Available online: http://ceur-ws.org/Vol-1196/cosecivi14_submission_23.pdf (accessed on 25 October 2019).
56. Paavilainen, J.; Alha, K.; Korhonen, H. Domain-specific playability problems in social networks games. *Int. J. Arts Technol.* **2015**, *8*, 4.
57. Desurvire, H.; Caplan, M.; Toth, J.A. Using heuristics to evaluate the playability of games. In Proceedings of the Extended Abstracts of the 2004 Conference on Human Factors and Computing Systems, Vienna, Austria, 24–29 April 2004; pp. 1509–1512.
58. Wang, J.-Y. Comparision of game experience and preferences between young and elderly. In Proceedings of the ICALIP 2014 International Conference on Audio, Language and Image Processing, Shanghai, China, 7–9 July 2014.
59. Smeddinck, J.; Gerling, K.; Tiemkeo, S. Visual complexity, player experience, performance and physical exertion in motion-based games for older adults. In Proceedings 15th Internatonal ACM SIGACCESS Conference on Computers and Accessibility, Bellevue, WA, USA, 21–23 October 2013.
60. Saenz-De-Urturi, Z.; Zapirain, B.G.; Zorrilla, A.M. Elderly user experience to improve a Kinect-based game playability. *Behav. Inf. Technol.* **2015**, *34*, 1040–1051, doi:10.1080/0144929x.2015.1077889.
61. Nawaz, A.; Skjaeret, N.; Ystmark, K.; Helbostad, J.L.; Vereijken, B.; Svanæs, D. Assessing seniors´user experience (UX) of exergames for balance training. In Proceedings of the NordiCHI'14, 8th Nordic Conference on Human-Computer Interaction: Fun, Fast, Foundational, Helsinki, Finland, 26–30 October 2014; pp. 578–587.
62. Marston, H. Design recommendations for digital game design within an ageing society. *Educ. Gerontol.* **2013**, *39*, 103–118

63. Pyae, A.; Raitoharju, R.; Luimula, M.; Pitkäkangas, P.; Smed, J. Serious games and active ageing: A pilot usability testing of existing games. *Int. J. Netw. Virtual Organ*. **2016**, *16*, 103–120
64. Altmeyer, M.; Lessel, P. The important of social relations for well-being change in old age—Do games preferences change as well? In Proceedings of the Positive Gaming: Workshop on Gamification and Games for Wellbeing (CHI PLAY'17), Amsterdam, The Netherlands, 15 October 2017.
65. Silva, P.A.; Holden, K.; Jordan, P.; Jordan, P. Towards a List of Heuristics to Evaluate Smartphone Apps Targeted at Older Adults: A Study with Apps that Aim at Promoting Health and Well-Being. In Proceedings of the 48th Hawaii International Conference on System Sciences, Kauai, HI, USA; 5–8 January 2015; pp. 3237–3246. doi: 10.1109/HICSS.2015.390.
66. Vasconcelos, A.; Silva, P.A.; Caseiro, J.; Nunes, F.; Teixeira, L. Designing Tablet-Based Games for Seniors: The example of CogniPlay, a Cognitive Gaming Platform; Fun and Games; Toulouse, France; September 2012. Available online : https://dl.acm.org/doi/10.1145/2367616.2367617 (accessed on 24 July 2019).
67. Laine, T.H. Mobile Educational Augmented Reality Games: A Systematic Literature Review and Two Case Studies. *Computers* **2018**, *7*, 19. doi:10.3390/computers7010019.
68. Staff CSDT. Intrinsic Motivation Inventary. Center for Self-Determination Theory. Available online: https://selfdeterminationtheory.org/intrinsic-motivation-inventory (accessed on 10 May 2019).
69. Del Valle, M.; Matos, L.; Díaz, A.E.; Perez, M.V.; Morales, J.R.V. Propiedades psicométricas escala satisfacción y frustración necesidades psicológicas (ESFNPB) en universitarios chilenos. *Propósitos Represent.* **2018**, *6*, 301–350, doi:10.20511/pyr2018.v6n1.202.
70. CSDT. Aspirations Index. Center for Self-Determination Theory. Available online: https://selfdeterminationtheory.org/aspirations-index/ (accessed on 22 May 2019).
71. Teixeira C., T.; Ishitani, L.; Vieira, N. Mobile game design for the elderly: A study with focus on the motivation to play. *Comput. Hum. Behav.* **2015**, *51*, 96–105, doi:10.1016/j.chb.2015.04.026.
72. Ibrahim, A.; Gutierrez, F.; González Sánchez, J.L.; Padilla-Zea, N. Educational Playability: Analyzing Player Experiences in Educational Video Games. In Proceedings of the ACHI 2012: The Fifth Internat. Conference on Advances in Computer-Human Interactions, Valencia, España, 30 January–4 February 2012.
73. González, S.J.; Gutiérrez, F. Jugabilidad como Medida de Calidad en el Desarrollo de Videojuegos. Available online: http://ceur-ws.org/Vol-1196/cosecivi14_submission_23.pdf (accessed on 10 July 2019).
74. De Vries, A.W.; Van Dieën, J.H.; Abeele, V.V.D.; Verschueren, S. Understanding Motivations and Player Experiences of Older Adults in Virtual Reality Training. *Games Heal. J.* **2018**, *7*, 369–376, doi:10.1089/g4h.2018.0008
75. Fanfarelli, J.; McDaniel, R. and Crossley, C. Adapting UX to the design of healthcare games and applications. *Entertain. Comput*. **2018**, *28*, 21–31.
76. Korn, O.; Tietz, S. Strategies for Playful design when gamifying rehabilitation: A study on user experience; 10th International Conference on Pervasive Technologies Related to Assistive Environments (PETRA'17), Island of Rhodes, Greece, 21–23 June, 2017.
77. Liukkonen, T.; Ahtosalo, H.; Heinonen, T.; Raitoharju, R.; Pitkakangas, P.; Makila, T. Motion Tracking exergames for elderly users. *IADIS Int. J. Comput. Sci. Inf. Syst.* **2015**, *10*, 52–64.
78. Koivisto, A.; Merilampi, S.; Sirkka, A. Mobile rehabilitation games—User experience study. In Proceedings of the European Conference on Games-Based Learning, Berlin, Germany, 9–10 Ocotober 2014.
79. Segura, E.M.; Waern, A; Segura, L.M.; Recio, D.L. Playification: The PhySeEr case. In Proceedings of the 2016 Annual Symposium on Computer-Human Interaction in Play (CHIPLAY'16), Austin, TX, USA, 16–19 October 2016; pp. 376–388. doi: 10.1145/2967934.2968099.
80. Merilampi, S.; Koivisto, A.; Virkki, J. Activation game for older adults—Development and initial user experiences. In Proceedings of the 6th Internat. Conference on Serious Games and Applications for Health SeGAH, Vienna, Austria, 16–18 May 2018.
81. Patsoule, E.; Koutsabasis, P. Redesigning web sites for older adults: A case study. *Behav. Inf. Technol.* **2014**, *33*, 516–573.
82. Pyae, A.; Luimula, M.; Smed, J. Investigating the usability of interactive physical activity games for elderly: A pilot study. In Proceedings of the IEEE 6th Conference on Cognitive Info-communications, Győr, Hungary, 19–21 October 2015.

83. Pham, T.P.; Theng, Y.L. Game controllers for older adults. In Proceedings of the International Conference on Distributed Smart Cameras, Hong Kong, China, 30 October–2 November 2012, pp. 284–285.
84. Nawaz, A.; Skjæret, N.; Helbostad, J.L.; Vereijken, B.; Boulton, E.; Svanaes, D. Usability and acceptability of balance exergames in older adults: A scoping review. *Heal. Informa. J.* **2016**, *22*, 911–931, doi:10.1177/1460458215598638.
85. Wagner, I.; Minge, M. The Gods Play Dice Together: The Influence of Social Elements of Gamification on Seniors' User Experience. In *HCI International 2015. Posters' Extended Abstracts, Part I*; Springer International Publishing: Berlin, Germany, 2015; pp. 334–339.
86. Boletsis, C.; McCallum, S. Evaluating a gaming system for cognitive screening and sleep duration assessment of elderly players: A pilot study. In Proceedings of the GALA 2016—LNCS 2016, Utrecht, The Netherlands, 5–7 December 2016; pp. 107–119. doi: 10.1007/978-3-319-50182-6_10.
87. Kappen, D.; Mirza-Babaei, P.; Nacke, L. Gamification of Older Adults' Physical Activity: An Eight-Week Study. Available online: http://hdl.handle.net/10125/50036 (accessed on 10 July 2019).
88. Genaro, L.; Vigouroux, N.; Gorce, P. Drag-and-drop for older adults using Touchscreen devices: Effects of screen sizes and interaction techniques on accuracy. In Proceedings of the IHM´14 26th Conférence Francophone sur l´Interaction Homme-Machine, Lille, France, 28–31 October 2014; pp. 139–146.
89. Aleem, S.; Capretz, L.; Ahmed, F. A consumer perspective on digital games. *IEEE Consum. Electron. Mag.* **2018**, *7*, 56–61. doi: 10.1109/MCE.2017.2714419.
90. Gerling, K.; Schulte, F. & Masuch, M. : Designing and evaluation digital games for frail elderly persons. In Proceedings of the 8th Internat. Conference on Advances in Computer Entertainment Technology, Lisbon, Portugal, 8–11 November 2011.
91. McLaughlin, A.; Bryant, M.; Sprufera, J.F.; Allaire, J.C.; Gandy, M. Usability an important goal for the design of therapeutic games for older adults. In Proceedings of the 10th International Conference on Engineering Psychology and Cognitive Ergonomics: Applications and Services, Las Vegas, NV, USA, 21–26 July 2013; pp. 358–364.
92. Awad, M., Fergurson, S. & Craig, C. Designing games for older adults: An affordance based approach. In Proceedings of the 2014 IEEE International Conference on Serious Games and Applications for Health (SeGAH), Rio de Janeiro, Brazil, 14–16 May 2014. doi: 10.1109/SeGAH.2014.7067103.
93. Diaz-Orueta, U.; Facal, D.; Nap, H.H.; Ranga, M.-M. What Is the Key for Older People to Show Interest in Playing Digital Learning Games? Initial Qualitative Findings from the LEAGE Project on a Multicultural European Sample. *Games Heal. J.* **2012**, *1*, 115–123, doi:10.1089/g4h.2011.0024.
94. Palacio, R.R.; Acosta, C.O.; Cortez, J.; Morán, A. Usability perception of different video game devices in elderly users. *Univ. Access Inf. Soc.* **2015**, *16*, 103–113, doi:10.1007/s10209-015-0435-y.
95. Mainza, E. HCI: Design Guidelines of Mobile Device Games for the Elderly. Master's Thesis, Malmo University, Malmö, Sweden, 2014.
96. Kawamoto, A.L.S.; Martins, V.F.; Da Silva, F.S.C. Usability Evaluation of an Application Designed for the Older Adults. In Proceedings of the Computer Vision, Sao Paulo, Brazil, 16–18 October 2013; pp. 189–192. doi:10.1007/978-3-642-41106-9_28.
97. Blat, J.; lluis, J.; Sayago, S. WorthPlay: Juegos Digitales Para un Envejecimiento Activo y Saludable. Available online: http://www.fgcsic.es/lychnos/es_ES/articulos/WorthPlay-juegos-digitales-para-un-envejecimiento-activo-y-saludable (accessed on 15 October 2019).
98. Cámara, A. El Juego en las Personas Mayores: Una vía de desarrollo personal. *Rev. Port. Pedag.* **2012**, *46*, 37–56.
99. Cabrera, M.E.F.; Ferrer, M.C.; Berenguer, M.T.R.; Puente, R.P. Juego como promoción de un envejecimiento saludable: Definición del usuario y pautas para el diseño de producto accessible. *Rev. Esp. Geriatr. Gerontol.* **2006**, *41* (Suppl. S2), 17–24.
100. Sopanen, J. Elderly Clients´Experiences of Playing a Video-Game. Bachelor's Thesis, Laurea University of Applied Sciences, Otaniemi, Finland, 2015.
101. Al Mahmud, A.; Mubin, O.; Shahid, S.; Martens, J.-B. Designing and evaluating the tabletop game experience for senior citizens. In Proceedings of the 5th Nordic Conference, New York, NY, USA, 1 January, 2008.

102. Nacke, L.; Nacke, A.; Lindley, C.A. Brain Training for Silver Gamers: Effects of age and game form on effectiveness, efficiency, self-assessment, and gameplay experience. *Cyberpsychol. Behav*. **2009**, *12*, 439–449.
103. Brown, J.A. Let's play. In Proceedings of the International Conference on Distributed Smart Cameras, Hong Kong, China, 30 October–2 November 2012.
104. Nacke, L.;. Ambinder, M.; Nacke, L.; Ambinder, M.; Canossa, A.; Mandryk, R.; Stach, T. Game metrics & biometrics: The Future of Player Experience Research In Proceedings of the Future Play, Vancouver, DC, Canada, 12–13 May 2009.
105. Al Mahmud, A.; Shahid, S.; Mubin, O. Designing with and for Older Adults: Experience from Game Design. *Comput. Inf. Sci.***2012**, *396*, 111–129, doi:10.1007/978-3-642-25691-2_5.
106. Pyae, A.; Liukkonen, T.N.; Mika, L.; Kattimeri, C.; Cauberghe, V.; Smed, J. Investigating the Finnish Elderly People's Attitudes and Motivation towards Digital Game-Based Physical Exercises. *Finn. J. eHeal. eWelf.* **2017**, *9*, 265–283, doi:10.23996/fjhw.60518.
107. Brown, J.; Zhou, J.; Salvendy, G. Exploring the Next Generation of Older Gamers: Middle-Aged Gamers. In *Human Aspects of IT for the Aged Population. Healthy and Active Aging: Second International Conference, ITAP 2016, Held as Part of HCI International 2016 Toronto, ON, Canada, July 17–22, 2016, Proceedings, Part II*; Springer Science and Business: Berlin, Germany, 2016; pp. 308–318. doi:10.1007/978-3-319-39949-2_30.
108. Shahid, S.; Mubin, O.; Al Mahmud, A. RACE: Towards Exploring the Design Dimensions of a Route Assisting and Communicating System for Elderly. In *Universal Access in Human-Computer Interaction. Addressing Diversity: 5th International Conference, UAHCI 2009, Held as Part of HCI International 2009, San Diego, CA, USA, July 19-24, 2009. Proceedings, Part I*; Springer Science and Business Media: Berlin, Germay, 2009; pp. 288–296.
109. Novick, D.; Vicario, J.; Santaella, B.; Gris, I. Empirical Analysis of Playability vs. Usability in a Computer Game. *Intell. Tutoring Systs* **2014**, *8518*, 720–731, doi:10.1007/978-3-319-07626-3_68.
110. Caroux, L.; Isbister, K.; Le Bigot, L.; Vibert, N. Player–video game interaction: A systematic review of current concepts. *Comput. Hum. Behav.* **2015**, *48*, 366–381, doi:10.1016/j.chb.2015.01.066.
111. Awad, M.; Craig, C. Player´s performance in cross generational game playing. In Proceedings of the Third Joint Internat. Conference, Seoul, Korea, 28–30 November 2017; pp. 23–24.
